# Exploring the Etiologies of Acquired Unilateral Proptosis: A Literature Review with Case Presentations

**DOI:** 10.1055/a-2719-8908

**Published:** 2026-01-22

**Authors:** Nam-Kyu Lim

**Affiliations:** 1 Department of Plastic & Reconstructive Surgery, Dankook University College of Medicine, Cheonan, Chungnam, Republic of Korea

**Keywords:** proptosis, exophthalmos, etiology

## Abstract

Acquired unilateral proptosis is a clinically significant condition with diverse etiologies, including trauma, inflammation or infection, tumors, and hemodynamic complications. This study aimed to improve recognition by integrating a literature review with illustrative case reports. Four representative cases were described, covering orbital infection, traumatic hematoma, carotid–cavernous fistula, and metastatic tumor. A focused literature review of publications from 2020 to 2024 was conducted, identifying 338 relevant studies, of which 171 met the inclusion criteria. Among the 171 eligible studies analyzed, tumors were the most frequent cause (93/171, 54.4%), followed by hemodynamic disorders (24/171, 14.0%), thyroid-related ophthalmopathy (13/171, 7.6%), infection, and inflammation. In tumors, metastatic tumor ( *n* = 10) represented the most common subtype, followed by sarcoma ( *n* = 9), retinoblastoma ( *n* = 7), and lymphoma ( *n* = 7). The four clinical cases aligned with these categories and illustrated a spectrum of outcomes—from irreversible vision loss in infection to visual preservation through timely endovascular intervention. This study provided a more detailed understanding of the diverse etiologies of acquired unilateral proptosis, suggesting the necessity of a multidisciplinary approach. Integrating these findings into clinical practice is expected to enhance early recognition, optimize treatment strategies, and ultimately improve patient outcomes.

## Introduction

Proptosis, characterized as the forward displacement and protrusion of one or both eyes, can vary in duration and severity, with some cases resulting in visual impairment. 1 Proptosis can be classified as axial, when the underlying pathology is located within the extraocular muscle cone (intraconal), or nonaxial, when the abnormality is situated outside this region (extraconal). 2 Given its association with both vision-threatening and life-threatening conditions, prompt evaluation and appropriate management are essential. Although comprehensive global epidemiologic data on proptosis are limited—given that it is a presenting symptom rather than a singular diagnosis—existing studies indicate that it is among the most common initial signs of orbital pathology. In orbital tumor cohorts, proptosis has been reported as the predominant presenting feature, with its frequency ranging from 19.0 to 84.6%, depending on tumor type and study population. 3 In thyroid eye disease, the presence and severity of proptosis are closely correlated with disease progression. 4

Accordingly, acquired unilateral proptosis presents a wide spectrum of etiologies, necessitating diverse treatment approaches based on the underlying cause. The four cases managed by the author also exhibited different etiologies of unilateral proptosis. In this study, these cases were presented alongside a literature review from the past 5 years, structured according to the classification proposed by Turnbull et al., which categorizes the causes into seven groups. 2 By providing a comprehensive overview, this study aimed to enhance awareness of etiological diversity of acquired unilateral proptosis, facilitating its application in clinical practice and underscoring its clinical significance.

## Materials and Methods

### Case Presentation

The following four cases of acquired unilateral proptosis encountered by the author were described. While proptosis improved with appropriate treatment, unfortunately, some cases resulted in complications such as permanent vision loss. The study was approved by the Institutional Review Board of our hospital (IRB number: 2019-07-018) and performed in accordance with the principles of the Declaration of Helsinki. Written informed consent for clinical photography was obtained from all patients.

**Fig. 1:**
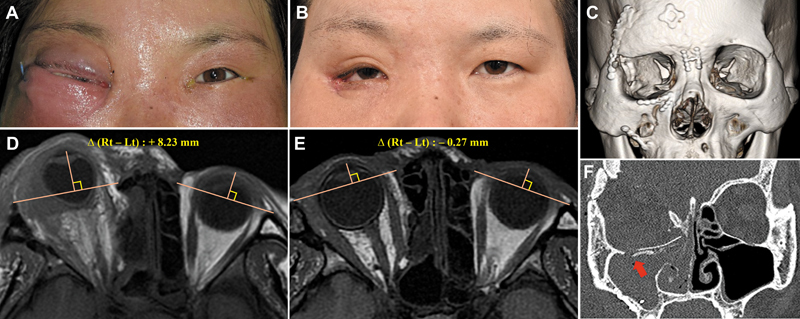
Preoperative and postoperative clinical photographs and radiological images of the patient in Case 1. ( **A, D** ) Preoperative images showing 8.23 mm of proptosis in the affected eye compared with the contralateral side on MRI exophthalmos measurement. ( **B, E** ) Postoperative images at 6 months, with MRI exophthalmos measurement showing −0.27 mm, indicating normalization. ( **C, F** ) Preoperative CT images revealing multiple fixation implants from prior surgery for severe facial fractures. The patient exhibited vulnerability to a sinus–orbit–brain connection, with severe maxillary sinusitis (red arrow) suspected to have contributed to the intraorbital infection. CT, computed tomography; MRI, magnetic resonance imaging.

**Fig. 2:**
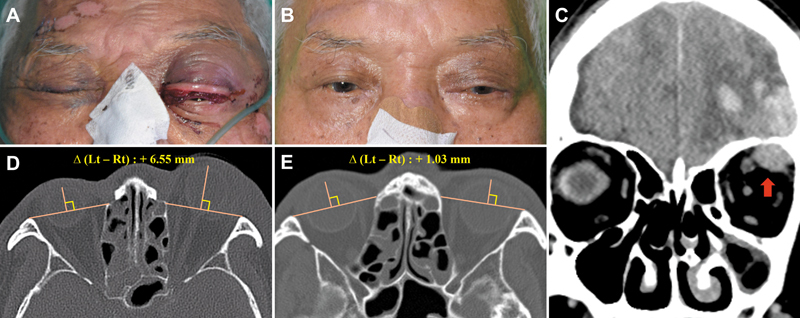
Preoperative and postoperative clinical photographs and radiological images of the patient in Case 2. ( **A, D** ) Preoperative images showing 6.55 mm of proptosis in the affected eye compared with the contralateral side on CT exophthalmos measurement. ( **B, E** ) Postoperative images at 5 months, with CT exophthalmos measurement showing 1.03 mm, indicating normalization. ( **C** ) Preoperative CT image revealing a hematoma within the orbit (red arrow), originating from a brain hemorrhage that had extended through the fractured orbital roof. CT, computed tomography.

**Fig. 3:**
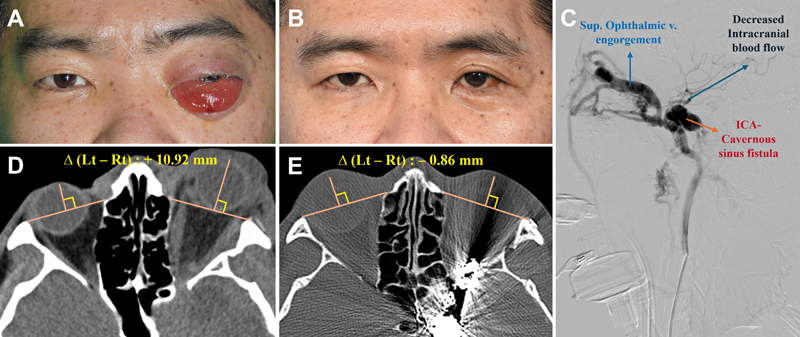
Preoperative and postoperative clinical photographs and radiological images of the patient in Case 3. ( **A, D** ) Preoperative images showing 10.92 mm of proptosis in the affected eye compared with the contralateral side on CT exophthalmos measurement. ( **B, E** ) Posttreatment images two months after endovascular embolization, with CT exophthalmos measurement showing −0.86 mm, indicating normalization. ( **C** ) Transfemoral cerebral angiography confirming superior ophthalmic vein engorgement due to carotid–cavernous fistula. CT, computed tomography.

**Fig. 4:**
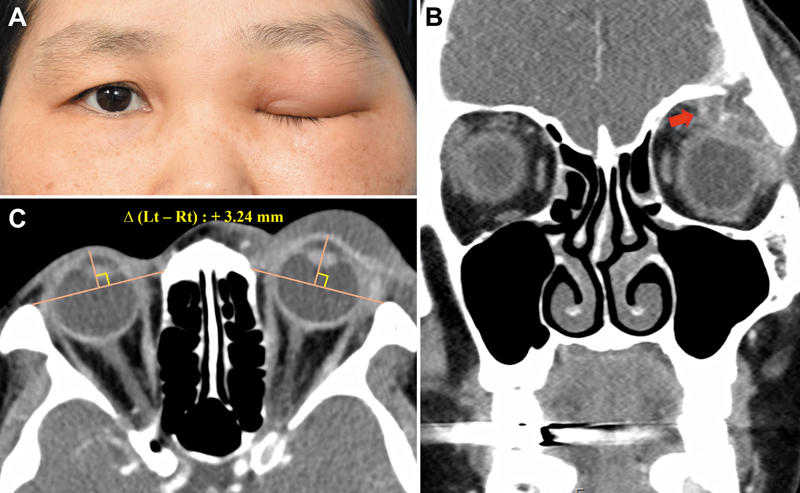
Preoperative clinical photograph and radiological images of the patient in Case 4. ( **A, C** ) Preoperative images showing 3.24 mm of proptosis in the affected eye compared with the contralateral side on CT exophthalmos measurement. ( **B** ) Preoperative enhanced CT scan revealed a mass-like lesion with orbital roof erosion. Postoperatively, the lesion was diagnosed as a metastatic tumor, and the patient was transferred to another hospital for chemotherapy, making long-term follow-up unavailable. CT, computed tomography.

*
A. Case 1: A 33-year-old female developed delayed intraorbital infection 6 years after craniofacial surgery for a traffic accident. She presented with progressive periorbital swelling and unilateral proptosis. Orbital computed tomography (CT) demonstrated +8.23 mm proptosis before treatment, related to severe maxillary sinusitis, and retained fixation implants. The presumed infection pathway was sinus–orbit communication along the implant surfaces. Despite multiple drainage procedures, implant removal, and prolonged systemic antibiotics, irreversible vision loss occurred. Follow-up CT confirmed resolution of proptosis (−0.27 mm) ( Fig. 1 ).
*
*
B. Case 2: An 80-year-old male developed unilateral proptosis after a traffic collision causing a frontal cerebral hemorrhage and orbital roof fracture. Unilateral proptosis developed 5 days after the trauma, and CT showed +6.55 mm proptosis preoperatively. Intraoperatively, an intraorbital hematoma extending through the fractured orbital roof was identified. The patient underwent hematoma evacuation and orbital roof reconstruction, after which residual proptosis decreased to +1.03 mm on follow-up CT. Fortunately, visual acuity was fully preserved ( Fig. 2 ).
*
*
C. Case 3: A 37-year-old male with LeFort I/II and sphenoidal sinus fractures developed unilateral proptosis 6 weeks after trauma. Initial CT demonstrated +10.92 mm proptosis. Cerebral angiography confirmed a carotid–cavernous fistula, and endovascular coiling was performed by neurosurgery. Posttreatment CT showed resolution of proptosis (−0.86 mm). His visual acuity was intact after treatment ( Fig. 3 ).
*
*
D. Case 4: A 41-year-old female with a history of breast cancer presented with unilateral proptosis. Orbital CT revealed +3.24 mm proptosis preoperatively, and intraoperative findings demonstrated an intraconal mass with orbital bony erosion. Histopathology confirmed metastatic carcinoma. The lesion was excised, but the patient was transferred for systemic oncological care and lost to follow-up. Vision was maintained until the last available examination ( Fig. 4 ).
*

### Literature Review

**Fig. 5:**
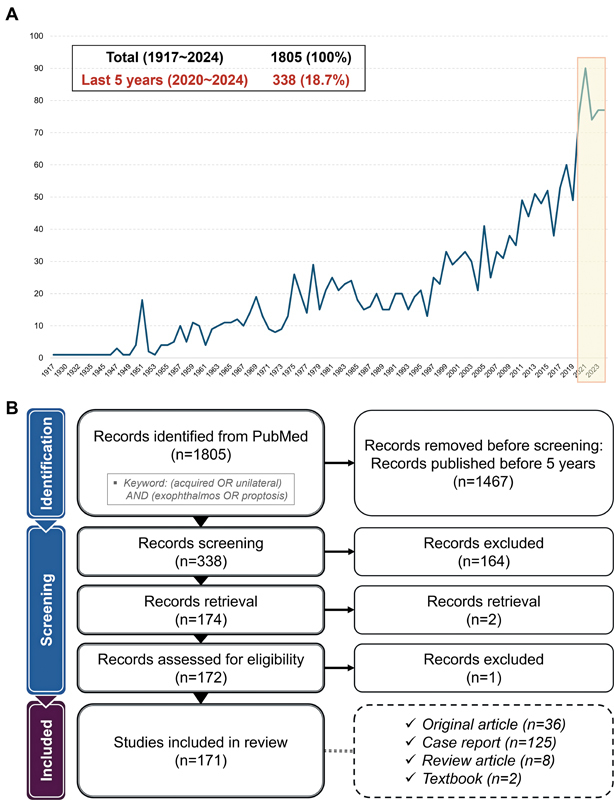
( **A** ) The results of a PubMed search using the keywords “(acquired OR unilateral) AND (exophthalmos OR proptosis)” were presented. A total of 1,805 articles were identified from 1917 to 2024, with 338 articles (18.7%) published in the most recent 5-year period (January 2020–December 2024). ( **B** ) The study selection process, illustrated according to the PRISMA 2020 flow diagram, showing records identified, screened, excluded, and included in the review. After screening, a total of 171 articles were included in the final review from the initial 338 retrieved articles.

To achieve a comprehensive understanding of the etiologies of acquired unilateral proptosis, a systematic literature review was conducted using PubMed for articles published between January 1, 2020, and December 31, 2024. The search keyword was “(acquired OR unilateral) AND (exophthalmos OR proptosis),” yielding a total of 338 studies ( Fig. 5A ). Following a rigorous screening process, 164 studies were excluded due to irrelevant titles or abstracts, and an additional 3 records were removed due to retrieval or eligibility issues, resulting in 171 articles included for analysis Supplementary Table 1 . Inclusion criteria were human studies reporting that acquired unilateral proptosis with an identifiable etiology, regardless of study type. Exclusion criteria were congenital or bilateral-only proptosis, editorials or letters without patient data, conference abstracts without sufficient data, and duplicate reports, for which the most complete version was retained. The study selection process was illustrated in Fig. 5B , which follows the PRISMA 2020 flow diagram format.

Regardless of article type, each study was assessed based on its described etiology, with each reported cause counted as a single causative factor. The identified etiologies of proptosis were systematically classified into seven major categories: 2 thyroid-related, trauma, tumor, infection, inflammation, hemodynamic disorders, and miscellaneous causes.

## Results

**Fig. 6:**
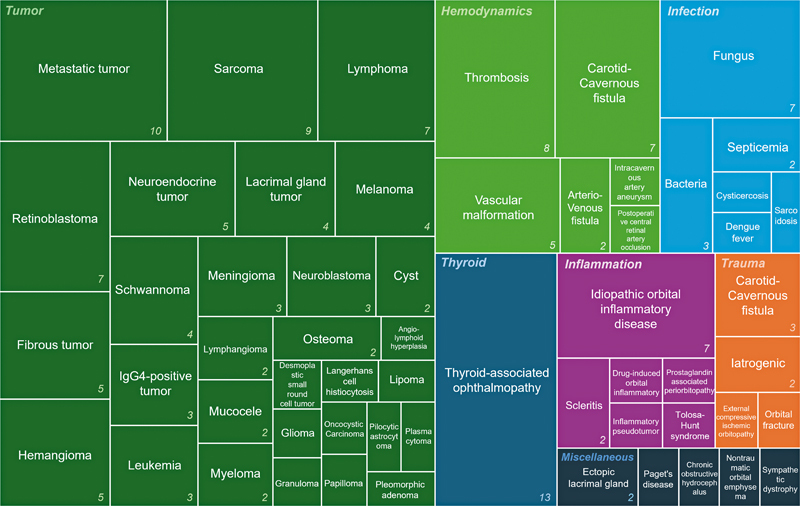
Etiological classification of 171 articles on acquired unilateral proptosis. A treemap visualization categorizing the studies into seven groups, with each study assigned to one primary etiology.

**Fig. 7:**
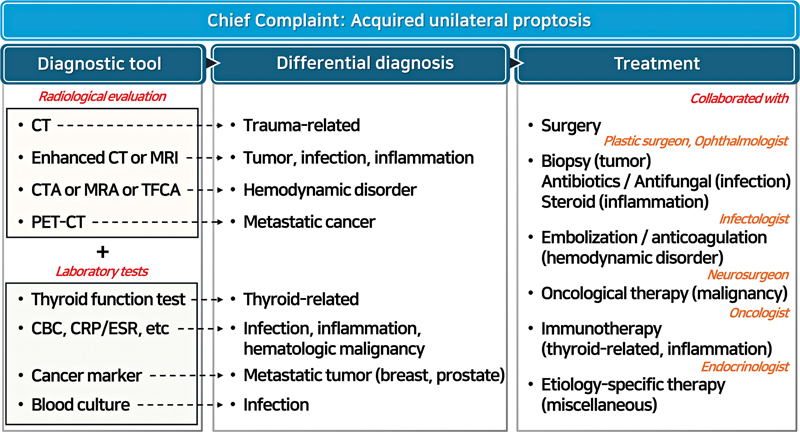
Diagnostic algorithm for acquired unilateral proptosis. Radiological and laboratory evaluations guide the main differential diagnoses, with representative treatment options summarized for clinical application.

Among the seven etiological categories, tumors accounted for the largest proportion of acquired unilateral proptosis cases (93/171, 54.4%). A total of 30 different tumor types were identified, with metastatic tumors being the most common ( *n* = 10), followed by sarcoma ( *n* = 9), retinoblastoma ( *n* = 7), and lymphoma ( *n* = 7). Among infectious causes, fungal infections were the most frequently observed ( *n* = 7). In the inflammatory category, which included six different conditions, idiopathic orbital inflammatory disease was the most prevalent, with 7 cases reported. Thyroid-related ophthalmopathy was reported in 13 cases. Interestingly, hemodynamic disorders represented the second most common etiology among the seven categories (24/171, 14.0%). A more detailed analysis revealed that orbital thrombosis ( *n* = 8) was the most frequently reported vascular event, followed by carotid–cavernous fistula ( *n* = 7; Fig. 6 ) To complement this etiological overview, a structured diagnostic algorithm was presented to assist clinical decision-making. The algorithm summarizes the typical diagnostic pathway, from radiological and laboratory evaluation to the major categories of differential diagnosis, and outlines representative treatment options for each ( Fig. 7 ).

## Discussion

The earliest reported case of acquired unilateral proptosis associated with the given keyword dates back to 1917, documenting a case of ethmoid osteoma. 5 Since then, numerous reports have described proptosis caused by thyroid-related conditions, tumors, and inflammatory diseases. However, due to the diagnostic limitations of imaging for intraconal lesions, early studies primarily focused on clinical symptoms and surgical exploration. 6 Efforts to develop noninvasive diagnostic methods for intraconal pathologies continued, and by the 1960s, contrast-enhanced X-ray and ultrasound were utilized to identify the underlying causes of proptosis. 7 8 With the widespread adoption of CT and magnetic resonance imaging (MRI) in the 1970s and 1980s, nonsurgical diagnosis of intraconal lesions became significantly more accessible, leading to a substantial increase in research and clinical studies on proptosis. Accordingly, the diverse etiologies of acquired unilateral proptosis have become increasingly evident over the years.

Identifying the underlying etiology is essential, as treatment approaches vary significantly depending on the cause. Delayed diagnosis or inappropriate management may result in irreversible optic nerve damage and vision loss. 1 Therefore, when evaluating a patient with proptosis, clinicians must carefully consider how to rapidly and efficiently establish an accurate diagnosis to ensure prompt and appropriate intervention. The initial step in evaluating a patient with proptosis is a thorough physical examination. If the proptosis is pulsatile, potential causes to consider include congestive cardiac failure, aortic regurgitation, and arteriovenous malformations. In such cases, the CT angiography should be considered as a crucial diagnostic tool to rule out. 9 And, given the deep anatomical location of the orbit and surrounding structures, CT and MRI are generally recommended for diagnostic evaluation. CT imaging is particularly useful for detecting bony abnormalities and acute orbital compartment syndrome. A key radiologic finding to recognize is the “guitar pick sign,” which indicates acute increased intraocular pressure. This sign is characterized by a posterior globe angle of less than 120 degrees on CT, a finding that correlates with a poor prognosis and an increased risk of permanent vision loss. 10 When this occurs, immediate decompressive procedures such as lateral canthotomy should be performed. Additionally, MRI can provide superior visualization of intraorbital soft tissue injuries, including encephalocele and optic neuropathy. However, its limitations include longer scan times, reduced sensitivity to bony fragments and potential risks in patients with ferromagnetic foreign bodies. 10

Thyroid function tests should not be overlooked in the diagnostic evaluation, as thyroid-related ophthalmopathy represents one of the leading causes of orbital inflammation. Its occurrence is estimated to range between 0.6 and 1.3 cases per 100,000 individuals annually. 11 While its exact pathophysiology remains unclear, proposed mechanisms include apical compression from enlarged extraocular muscles or fat, ischemia due to increased retrobulbar pressure, and perineural inflammation. 11 12 Idiopathic orbital inflammation (IOI) is a benign, noninfectious, and nonspecific inflammatory condition of the orbit. It is the third most common orbital disease in adults, following thyroid-related ophthalmopathy and lymphoproliferative disorders. 13 Recent advancements in scientific research have led to increased investigation into the underlying pathogenesis of IOI. Two predominant theories have emerged: the autoimmune hypothesis and the viral infection hypothesis. While immune-mediated mechanisms have gained broader acceptance, a comprehensive and definitive pathophysiological explanation has yet to be established. 13

In addition, in patients with a history of trauma or prior orbital surgery, potential complications such as delayed hemorrhage from implants or posttraumatic encephalocele could be considered. 9 14 Notably, pediatric patients with orbital roof fractures demonstrating over 2-mm diastasis and associated frontal cerebral contusion may have an increased risk of developing intraorbital encephalocele. 15 Another uncommon but serious complication is orbital involvement following subgaleal hematoma. It results from blood accumulation under pressure at the superior orbital ridges, particularly in children, where the galea aponeurosis and periosteum attachments are loosely connected. The hematoma can dissect and detach the arcus marginalis from the orbital rim, leading to subperiosteal blood accumulation, which manifests as proptosis and decreased vision. Since coagulopathies can predispose patients to subgaleal hematoma, a thorough hematologic evaluation should be performed, including assessments of vitamin K levels, fibrinogen concentration, clotting factor deficiencies, and platelet disorders. 16

Compared with previous articles, this review analysis underscores the predominance of tumors (54.4%) among acquired unilateral proptosis, while also quantifying other categories such as thyroid-associated disease, infection, trauma, and hemodynamic disorders. This highlights not only the relative frequency of major etiologies but also the diversity of underlying causes. The four representative cases serve as illustrative examples of the review findings. Case 1 demonstrated the vision-threatening course of orbital infection, echoing the poor visual outcomes described among infectious etiologies in the review. Case 2 illustrated traumatic orbital roof fracture with hematoma, reinforcing the importance of rapid intervention to preserve vision. Case 3 highlighted a hemodynamic disorder—carotid–cavernous fistula—emphasizing the critical role of timely endovascular intervention and interdisciplinary collaboration. Case 4 exemplified metastatic orbital disease, relating to the 10 metastatic tumors detected in the pooled analysis and reinforcing the need to consider systemic malignancy in new-onset unilateral proptosis. Collectively, these cases bridge the systematic data with real-world clinical practice, underscoring the necessity of tailored diagnostic and therapeutic approaches.

Conversely, very rare entities such as Erdheim–Chester disease, 17 hydatid disease, 18 or dengue-related orbital involvement, 19 while clinically intriguing, are worth acknowledging because they underscore the wide spectrum of potential etiologies. Even if their frequency is extremely low, awareness of such conditions may broaden the clinician's differential diagnosis and prevent overlooking rare but important causes of proptosis.

While our study incorporated the latest research, one limitation was the potential overrepresentation of rare case reports due to the nature of recently published literature. Additionally, diseases from original studies and case reports were counted equivalently, which may have introduced bias in the relative frequency of different etiologies. Moreover, this review relied primarily on PubMed, which provides comprehensive biomedical coverage; however, relevant articles indexed exclusively in other databases such as Embase or Scopus may not have been captured. Nevertheless, this study provided a more detailed understanding of the diverse etiologies of acquired unilateral proptosis, and these findings may indicate the need for a multidisciplinary approach involving plastic surgeons, ophthalmologists, radiologists, neurosurgeons, and endocrinologists for accurate diagnosis and effective management. Such collaboration is highly recommended, particularly in complex cases requiring comprehensive evaluation and coordinated treatment.

In conclusion, this study presented case reports of acquired unilateral proptosis and explored its diverse etiologies. Integrating these findings into clinical practice may help enhance early recognition, optimize treatment strategies, and ultimately improve patient outcomes.
