# Supplementary material for: Exploring the Etiologies of Acquired Unilateral Proptosis: A Literature Review with Case Presentations
Source: Arch Plast Surg. 2026 Jan 22;53(2):164–71. doi: 10.1055/a-2719-8908 (PMC13030925; doi:10.1055/a-2719-8908)
Supplement: Supplementary file 1 — Supplementary Material [file 10-1055-a-2719-8908-s25apr0073rev.pdf]

Thieme

S1 table. Summary of the 171 included studies, presenting article type, etiology, and sample size.

No.	Author	Article type	Title	Etiology	Sample size	PMID
1	Yang M, He W.	Original	Age and gender influence on clinical manifestations of thyroid-associated ophthalmopathy: a case series of 2479 Chinese patients	TAO	2479	39421533
2	Lai KK, Ali Abdulla Aljufairi FM, Sebastian JU, et al.	Original	Euthyroid graves' ophthalmopathy in a Chinese population: A cross-sectional follow-up study	TAO	1266	38444235
3	Eshraghi B, Pourazizi M, Abbasi M, et al.	Original	A comparison between bilateral and unilateral thyroid eye disease	TAO	383	37067696
4	Yang M, Wang Y, Du B, et al.	Original	Clinical phenotypes of euthyroid, hyperthyroid, and hypothyroid thyroid-associated ophthalmopathy	TAO	2158	36806995
5	Bayarmunkh O, Ganbold C, Das S, et al.	Original	Association of clinical course with thyroid-stimulating immunoglobulin in Graves' ophthalmopathy in Mongolians	TAO	238	36395261
6	Alfutaisi A, Osman A, Al Siyabi ZS, et al.	Case report	Unilateral Graves' Orbitopathy in a Patient with Marine-Lenhart Syndrome: A case report	TAO	1	39634811
7	Smółka K, Perenc L, Pelc J, et al.	Case report	Thyrotoxic Myopathy with Nonspecific Ophthalmopathy in a Two-Year-Old Child: Case Report and Literature Review	TAO	1	39458129
8	Bhattarai HB, Thapaliya I, Dhungana S, et al.	Case report	Unilateral proptosis in a patient with thyroid eye disease: A case report	TAO	1	37533488
9	Das L, Singh U, Malhotra B, et al.	Case report	Thyroid eye disease with concurrent orbital lymphoma: a radiological surprise	TAO	1	35332876
10	Cavasin N, Presotto F, Bellamio M, et al.	Case report	Cavernous Sinus Dural Arteriovenous Fistula in a Patient with Thyroid-Associated Ophthalmopathy: Complete Resolution after Medical Treatment	TAO	1	35053788
11	El Othman R, Ephrem C, Touma E, et al.	Case report	A case report of thyroid-associated Orbitopathy with elevated TPO antibodies	TAO	1	33246456
12	Panagiotou G, Perros P.	Review	Asymmetric Graves' Orbitopathy	TAO	N/A	33391188
13	Shah SS, Patel BC.	Book	Thyroid Eye Disease	TAO	N/A	35881739
14	Saleem MS, Yadlapalli SS, Jamil S, et al.	Case report	Traumatic Carotid Cavernous Fistula Resulting in Symptoms in the Ipsilateral Eye: A Case Report	Trauma (CCF)	1	36465727
15	Zhang C, Tianjia Z, Lv H, et al.	Case report	The Possibility of Internal Carotid-Cavernous Fistula After Maxillary Fracture	Trauma (CCF)	1	36000766
16	Liang J, Xie X, Sun Y, et al.	Case report	Bilateral carotid cavernous fistula after trauma: a case report and literature review	Trauma (CCF)	1	34736536
17	Ghiam BK, Liu BJ, Echegaray JJ, et al.	Review	External Compressive Ischemic Orbitopathy: A Rising Clinical Entity	Trauma (ECIO)	46	37486344
18	Teoh RJJ, Ain Masnon N, Bahari NA, et al.	Case report	Acquired proptosis and progressive abducens nerve palsy due to overpacked coiling material: rare sequelae of endovascular treatment for carotid cavernous fistula	Trauma (Iatrogenic)	1	37816571

19	Enríquez AB, Wheelock-Gutierrez L, Golzarri MF, et al.	Original	Unilateral Orbital Emphysema Secondary to Vitreoretinal Surgery	Trauma (Iatrogenic)	16	32192937
20	Cammarata G, Altieri R, Certo F, et al.	Review	Post-traumatic intra-orbital meningoencephalocele in adults: technical note on a rare entity and review of the literature	Trauma (Orbital fracture)	29	36471011
21	Ahmed HS, Thrishulamurthy CJ, Venkatesh S, et al.	Review	Ophthalmic Manifestations of Angiolymphoid Hyperplasia with Eosinophilia: A Systematic Review and Pooled Analysis of 86 Cases	Tumor (Angiolymphoid hyperplasia)	86	39718142
22	Wilde C, Vahdani K, Thaung C, et al.	Original	Presenting features for developmental cysts of the orbit	Tumor (Cyst)	358	35064209
23	Attar A, Khademi B, Jalalpour MH.	Case report	Isolated hydatid cyst in the medial rectus muscle: unveiling a rare orbital occurrence	Tumor (Cyst)	1	39532786
24	Biswas J, Maity R, Dhali A, et al.	Case report	Multifocal Desmoplastic Small Round Cell Tumor: A Case Report of a Rare Neoplasm	Tumor (DSRCT)	1	38440042
25	Li J, Wang YC, Chen LX, et al.	Original	Clinical and pathological analysis of 35 cases of ocular adnexal solitary fibrous tumor	Tumor (fibrous tumor)	35	36860108
26	Rahmani M, Hendi K, Dalfardi S, et al.	Case report	Juvenile Psammomatoid Ossifying Fibroma of the Orbital Roof: A Rare Cause of Proptosis among Children	Tumor (fibrous tumor)	1	32756056
27	Ting XW, Sothiraghagan S, W Md Kasim WM, et al.	Case report	A Clinicopathologic Study of Seven Cases of Orbital Solitary Fibrous Tumours	Tumor (fibrous tumor)	7	32596077
28	Bertin H, Huon JF, Guillot P, et al.	Case report	Fibrous dysplasia of the orbital region: Series of 12 cases and review of the literature	Tumor (fibrous tumor)	12	32376037
29	Alabdulrazaq ES, Gurnani B.	Book	Orbital Solitary Fibrous Tumor	Tumor (fibrous tumor)	N/A	37983342
30	Panagopoulou P, Athanasiadis D, Anastasiou A, et al.	Original	Pediatric Optic Pathway Gliomas: A Report from Northern Greece	Tumor (Glioma)	23	37696004
31	Naggar A, Toufga Z, Ech-Cherif El Kettani N, et al.	Case report	Granulomatosis with polyangiitis presenting with unilateral exophthalmos: A case report	Tumor (Granuloma)	1	35663807
32	Vahdani K, Rose GE.	Original	Presenting Characteristics for Symptomatic, as Compared to Asymptomatic (assumed), Orbital Cavernous Venous Malformations	Tumor (Hemangioma)	414	35502799
33	Bi YW, Cai RR, Wang SY, et al.	Original	The clinicopathologic features and differential diagnosis of ocular Kimura disease and epithelioid hemangioma	Tumor (Hemangioma)	13	34865407
34	Meling TR, Steffen H.	Review	Cavernous hemangioma of the orbit: diagnosis and management	Tumor (Hemangioma)	N/A	33146966
35	Fabozzi GL, d'Avella E, Burroni M, et al.	Case report	Endoscopic transorbital eyelid approach for the removal of an extraconal cavernous venous malformation: Case report	Tumor (Hemangioma)	1	35937596
36	Oukassou R, Chadli J, Bouabbadi S, et al.	Case report	Unilateral proptosis secondary to cavernous hemangioma in a child: A case report	Tumor (Hemangioma)	1	32684323
37	Yang Y, Jin X, Xu M, et al.	Original	Clinical characteristics and treatment outcomes of patients with IgG4-positive ocular adnexal marginal zone B-cell lymphoma	Tumor (IgG4-positive tumor)	22	38767546

38	Yazici B, Onaran Z, Yalcinkaya U.	Case report	IgG4-Related Dacryoadenitis With Fibrous Mass in a 19-Month-Old Child: Case Report and Literature Review	Tumor (IgG4-positive tumor)	1	39136975
39	Zhang X, Wu G, Wang M, et al.	Review	Novel Advances in the Study of IgG4-Related Disease in the Eye and Ocular Adnexa	Tumor (IgG4-positive tumor)	N/A	35843209
40	Bohara D, Shrestha GB, Shrestha BMS.	Case report	A Case Report on Unilateral Non-axial Proptosis of a Young Female: Lacrimal Gland Tumour	Tumor (Lacrimal gland tumor)	1	38976341
41	Aucoin AJ, Chawla H, Holleman GT, et al.	Case report	Primary ductal adenocarcinoma of the lacrimal gland with sarcomatoid differentiation: case report and genomic analysis	Tumor (Lacrimal gland tumor)	1	37611021
42	Kim JS, Proia AD, Liss J, et al.	Case report	Multiple Cranial Neuropathies as the Initial Presentation of Primary Ductal Adenocarcinoma of the Lacrimal Gland	Tumor (Lacrimal gland tumor)	1	35030152
43	Topilow NJ, Stevens SM, Chen Y, et al.	Case report	A rare case of mucoepidermoid carcinoma ex pleomorphic adenoma of the lacrimal gland	Tumor (Lacrimal gland tumor)	1	34939520
44	Lakatos K, Sterlich K, Pötschger U, et al.	Original	Langerhans Cell Histiocytosis of the Orbit: Spectrum of Clinical and Imaging Findings	Tumor (LCH)	31	33157073
45	Nath U, Gupta J, Chakraborti C.	Case report	Unilateral Proptosis as the Initial Manifestation of Acute Myeloid Leukemia in a Young Adult	Tumor (Leukemia)	1	39628729
46	Schrijver IT, Kalikmanov-Mikhaylovskaya E, Sandberg Y.	Case report	Primary plasma cell leukaemia presenting as unilateral proptosis	Tumor (Leukemia)	1	38024621
47	Al-Mujaini A, Al-Shaaibi M, Al-Mughaizwi T, et al.	Case report	Unilateral Proptosis: A Rare Presenting Sign of Acute Myeloid Leukemia	Tumor (Leukemia)	1	35915765
48	Shrestha P, Shrestha GB.	Case report	Orbital Lipoma as an Uncommon Cause of Unilateral Proptosis: A Case Report	Tumor (Lipoma)	1	32982481
49	Kummari S, Ranga M.	Case report	Orbital Lymphangioma in an Adolescent Male: A Case Report and Review of Literature	Tumor (Lymphangioma)	1	39737324
50	Mai AP, Quigley EP 3rd, Harrie RP.	Case report	Acute Unilateral Proptosis After Blunt Orbital Trauma in an Adolescent Patient	Tumor (Lymphangioma)	1	36394845
51	Qi-Xian T, Chew-Ean T, Abdul Rahim A, et al.	Original	Orbital Tumours in Northern Malaysia: A Five-Year Review	Tumor (Lymphoma)	28	35004088
52	Vilarello BJ, Jacobson PT, Gudis DA, et al.	Case report	B-cell Lymphoblastic Lymphoma Presenting as a Sinonasal Mass: A Case Report	Tumor (Lymphoma)	1	38738089
53	Mani S.	Case report	Case Report: Conjunctival Chemosis as a Presenting Sign of Plasmablastic Lymphoma in an HIV-negative Patient	Tumor (Lymphoma)	1	37278658
54	Sharif MW, Mungara S, Bajaj K, et al.	Case report	Orbital Lymphoma Masquerading as Euthyroid Orbitopathy	Tumor (Lymphoma)	1	36925990
55	Saxena S, Koka K, Scott JX, et al.	Case report	Sporadic Burkitt Lymphoma Involving the Orbit - A Report of Two Cases and Review of Literature	Tumor (Lymphoma)	2	34978250

56	Qin VL, Briceño CA.	Case report	Systemic follicular lymphoma in a patient with a history of thyroid eye disease: a case report	Tumor (Lymphoma)	1	34579605
57	Yawar B, Malik Z, Naz F.	Case report	A rare case of orbital myositis	Tumor (Lymphoma)	1	33754537
58	Opgenorth TA, Gibson RMB, Mamaliger N, et al.	Case report	Unilateral invasive intraocular melanoma with choroidal and optic nerve involvement resulting in bilateral vision loss in a Labrador Retriever	Tumor (Melanoma)	1	37092364
59	Mathieu A, Nicot R, Schlund M.	Case report	A Recurrent Orbital Hemorrhage in an Older Adult	Tumor (Melanoma)	1	35951333
60	Narayanan N, Padwal U, Gopinathan I, et al.	Case report	Malignant melanoma of the rectum presenting as orbital metastasis	Tumor (Melanoma)	1	33120715
61	Adetunji MO, McGeehan B, Lee V, et al.	Review	Primary orbital melanoma: A report of a case and comprehensive review of the literature	Tumor (Melanoma)	88	32900269
62	Teng Siew T, Mohamad SA, Sudarno R, et al.	Case report	Unilateral Proptosis and Bilateral Compressive Optic Neuropathy in a Meningioma Patient	Tumor (Meningioma)	1	38455798
63	Alberta IB, Shen R, Tjahjadi M.	Case report	Unilateral proptosis as a neglected case of sphenoid wing meningioma: a case report and healthcare challenges in rural Indonesia	Tumor (Meningioma)	1	37333787
64	Yang JH, Li MS, Shen MJ, et al.	Case report	Unilateral Orbitopathy Caused by Skull Base Chordoid Meningioma	Tumor (Meningioma)	1	36899959
65	Zhao Y, Yu S, Mu M, et al.	Original	Clinical and Imaging Characteristics of Metastatic Orbital Tumours in North China	Tumor (Metastatic tumor)	36	38370869
66	Sharma G, Wanis M.	Case report	Unilateral proptosis: a rare presentation of metastatic prostate cancer	Tumor (Metastatic tumor)	1	38026747
67	Al Tawil L, Alkatan HM, Alnuman R, et al.	Case report	Unilateral acquired blepharoptosis due to orbital metastatic disease as an initial presentation of an overlooked breast carcinoma - A case report	Tumor (Metastatic tumor)	1	37666162
68	Chanda UL, Knapp C.	Case report	A rare case of metastatic paraspinal rhabdomyosarcoma presenting with proptosis in an adult patient	Tumor (Metastatic tumor)	1	36131600
69	Silver JA, Almhanedi H, Lai JK, et al.	Case report	A Case of Unilateral Proptosis Secondary to Prostatic Metastasis	Tumor (Metastatic tumor)	1	36007169
70	Mahyuddin M, Theresia K, Anggraini N, et al.	Case report	Orbital metastases as the initial clinical manifestation of thyroid carcinoma: A case series	Tumor (Metastatic tumor)	3	35388250
71	Montejano-Milner R, López-Gaona A, Fernández-Pérez P, et al.	Original	Orbital metastasis: Clinical presentation and survival in a series of 11 cases	Tumor (Metastatic tumor)	10	35152953
72	Ozer M, Juneja K, Mahdi M, et al.	Case report	Unilateral Proptosis due to Orbital Metastasis of Paratesticular Leiomyosarcoma	Tumor (Metastatic tumor)	1	33869092
73	Agarwal R, Khare A, Gupta N.	Case report	Isolated Unilateral Orbital Metastasis From Ewing Sarcoma Detected on FDG PET/CT Scan	Tumor (Metastatic tumor)	1	33630799
74	Karti O, Ozdemir O, Top Karti D, et al.	Case report	Orbital Metastasis Secondary to Breast Cancer: A Rare Cause of Unilateral External Ophthalmoplegia	Tumor (Metastatic tumor)	1	34188339
75	Vahdani K, Rose GE.	Original	Ophthalmic Presentation of Undiagnosed Sinonasal Masses	Tumor (Mucocele)	448	33237670
76	Gozgec E, Ogul H.	Case report	Unilateral Vision Loss Due to Isolated Onodi Cell Mucocele	Tumor	1	33003159

				(Mucocele)		
77	Xiong J, Tong JY, Hyer J, et al.	Original	Orbital Myeloma and Plasmacytoma: An Australian Study	Tumor (Myeloma)	21	39254970
78	Shoji MK, Chen Y, Topilow NJ, et al.	Original	Orbital Involvement in Multiple Myeloma	Tumor (Myeloma)	7	36661857
79	El Omri M, Njima MB, Mesbah L, et al.	Case report	Exophthalmos revealing an olfactory esthesioneuroblastoma: A case report	Tumor (Neuroblastoma)	1	38754157
80	Chen L, Wang J, Yang Z, et al.	Case report	Olfactory Neuroblastoma of the Sinonasal Tract with Prominent Orbital Protrusion: A Case Report and Literature Review	Tumor (Neuroblastoma)	1	36452830
81	Kumar P, Sundriyal D, Bhandari R, et al.	Case report	Facial disfigurement due to olfactory neuroblastoma: beauty regained with chemotherapy	Tumor (Neuroblastoma)	1	33029949
82	Arrivi G, Specchia M, Pilozzi E, et al.	Review	Diagnostic and Therapeutic Management of Primary Orbital Neuroendocrine Tumors (NETs): Systematic Literature Review and Clinical Case Presentation	Tumor (NET)	N/A	38397981
83	Qiao J, Wang Y, He W.	Case report	Neuroendocrine neoplasms of the orbit: report of three cases and a literature review	Tumor (NET)	3	37740637
84	Stephen MA, Ahuja S, Jayasri P, et al.	Case report	Peripheral primitive neuroectodermal tumor of the orbit in Graves' ophthalmopathy - A rare presentation	Tumor (NET)	1	36968771
85	Zhang Y, Li YY, Yu HY, et al.	Case report	Rare neonatal malignant primary orbital tumors: Three case reports	Tumor (NET)	3	34621833
86	Li Y, Chen L, Zhou X, et al.	Case report	A case report of neonatal orbital peripheral primitive neuroectodermal tumor and literature review	Tumor (NET)	1	32493126
87	Li J, Yang R, Liu R, et al.	Case report	Orbital Oncocytic Carcinoma: A Comprehensive Case Series and Literature Review	Tumor (Oncocystic carcinoma)	13	39028105
88	Movio G, Ahmed S.	Case report	Paranasal Osteoma: The Importance of Surveillance	Tumor (Osteoma)	1	37674766
89	Sendul SY, Mavi Yildiz A, Yildiz AA.	Case report	Giant osteomas: Clinical results and surgical approach from ophthalmic point of view	Tumor (Osteoma)	6	33008271
90	Sagar P, Singh I, Rajpurohit P, et al.	Case report	Inverted papilloma presenting as unilateral proptosis: A case report with review of literature	Tumor (Papilloma)	1	31920456
91	Homer NA, Epstein A, Durairaj VD, et al.	Case report	Acute post-partum vision loss due to pilocytic astrocytoma	Tumor (Pilocystic astrocytoma)	1	32875164
92	Permaisuari N, Anggraini N, Mahyuddin M, et al.	Case report	A Case Series of Aggressive Orbital Plasmacytomas	Tumor (Plasmacytoma)	2	36925734
93	Lygeros S, Tsapardoni F, Mastronikolis S, et al.	Case report	Pleomorphic adenoma of the maxillary sinus with orbital extension presenting with exophthalmos: a case report	Tumor (Pleomorphic adenoma)	1	34805758
94	Moin M, Malik TG, Siddiq L.	Original	Clinical Patterns And Outcomes Of Retinoblastoma In A Tertiary Care Centre Of A Developing Country	Tumor (Retinoblastoma)	47	37817703
95	Beniwal V, Maheshwari G, Beniwal S, et al.	Original	Retinoblastoma: A review of clinical profile at a regional cancer center in Northwest India	Tumor (Retinoblastoma)	54	36412421

96	Surukrattanaskul S, Keyurapan B, Wangtiraumnuay N.	Original	Correlation between clinical presentations, radiological findings and high risk histopathological features of primary enucleated eyes with advanced retinoblastoma at Queen Sirikit National Institute of Child Health: 5 years result	Tumor (Retinoblastoma)	33	35857757
97	Diatewa BM, Maneh N, Domingo AS, et al.	Original	Retinoblastoma at the Campus-University Teaching Hospital in Lomé, Togo, from 2014 to 2018	Tumor (Retinoblastoma)	75	35753853
98	Hordofa DF, Daba KT, Mengesha AA.	Original	Clinical Presentation of Retinoblastoma in Ethiopia: A Case of Jimma University Medical Center Pediatric Oncology Unit, Southwest Ethiopia	Tumor (Retinoblastoma)	36	35221612
99	Kaliki S, Jakati S, Vempuluru VS, et al.	Original	Retinoblastoma associated with orbital pseudocellulitis and high-risk retinoblastoma: a study of 32 eyes	Tumor (Retinoblastoma)	32	34363179
100	Zia N, Hamid A, Iftikhar S, et al.	Original	Retinoblastoma Presentation and Survival: A four-year analysis from a tertiary care hospital	Tumor (Retinoblastoma)	93	31933609
101	Li Y, Wang Y, He W.	Case report	Case report: Orbital myeloid sarcoma: a report of two rare cases and review of the literature	Tumor (Sarcoma)	2	39525664
102	Lucero KAJ, Woo KI.	Case report	Orbital Liposarcoma Progression From a Well-Differentiated to a Dedifferentiated Type in a Young Patient	Tumor (Sarcoma)	1	37721315
103	Nhung TH, Minh VL, Tuyet TT, et al.	Case report	Orbital rhabdomyosarcoma in a 19-year-old male patient: A case report and literature review	Tumor (Sarcoma)	1	37334324
104	Shrestha T, Mainali S, Poudel S, et al.	Case report	A rare case of pediatric orbital rhabdomyosarcoma in Nepal: a case report	Tumor (Sarcoma)	1	37113866
105	Rogelio PNA, Ranche FKT, Pe-Yan MR.	Case report	Hyperostosis in orbital rhabdomyosarcoma	Tumor (Sarcoma)	1	35725289
106	Manpreet S, Sagarika S, Pulkit R, et al.	Case report	Acute unilateral proptosis in childhood: suspect myeloid sarcoma	Tumor (Sarcoma)	2	33817441
107	Andrew D, Cicilet S, Shyam K, et al.	Case report	Elderly male patient with unilateral proptosis and decreased vision of the left eye	Tumor (Sarcoma)	1	33127716
108	Savino G, Petrone G, Volpe G, et al.	Case report	Vertical restrictive strabismus associated with proptosis: Similar clinical signs, different etiopathogenetic causes. A report of three patients	Tumor (Sarcoma)	3	32720817
109	Wang Y, Du B, Yang M, et al.	Case report	Paediatric orbital alveolar soft part sarcoma recurrence during long-term follow-up: a report of 3 cases and a review of the literature	Tumor (Sarcoma)	3	32085747
110	Parida S, Sachdeva V, Warkad VU.	Case report	Isolated cisternal third cranial nerve schwannoma: an insight into a clinico-radiological features	Tumor (Schwannoma)	1	39658235
111	Murofushi K, Tsutsumi S, Tomita S, et al.	Case report	Frontal nerve schwannoma in a 16-year-old girl presenting with a rapid growth for 2 years	Tumor (Schwannoma)	1	39640354
112	Uppal S, Saggar V, Scalia G, et al.	Case report	Unilateral orbital schwannoma arising from the supraorbital nerve: Report of a rare case	Tumor (Schwannoma)	1	38161625
113	Hötte GJ, Meijer N, Verdijk RM, et al.	Case report	Accelerated growth of orbital schwannomas during pregnancy does not correlate with sex hormone- or growth factor receptor status	Tumor (Schwannoma)	3	32264727
114	Davis E, Molina C, Kancharla A, et al.	Case report	A Rare Neonatal Infection: Methicillin-Resistant Staphylococcus aureus (MRSA) Orbital Cellulitis	Infection (Bacteria)	1	39192926
115	Huang Z, Zhou B.	Case report	Acute unilateral proptosis	Infection (Bacteria)	1	36958736
116	Toh ZYC, Cameron A.	Case report	Orbital compartment syndrome secondary to subperiosteal abscess initiated by	Infection	1	35332011

			barotrauma	(Bacteria)		
117	Maurya RP, Mishra CP, Roy M, et al.	Original	Ocular cysticercosis at a teaching hospital in Northern India	Infection (Cysticercosis)	36	34084028
118	Maan V, Guha S, Sapra H, et al.	Original	Ocular and adnexal manifestations post dengue hemorrhagic fever	Infection (Dengue fever)	13	39331441
119	Boiko NV, Kolesnikov VN, Khanamirov AA, et al.	Original	COVID-19 associated sino-orbital mucormycosis	Infection (Fungal)	13	37184552
120	Mittal A, Mahajan N, Pal Singh Dhanota D, et al.	Original	SARS-CoV-19-associated Rhino-orbital and cerebral mucormycosis: clinical and radiological presentations	Infection (Fungal)	36	36029277
121	Abdelsamie AM, Abdelazim HM, Elnems MG, et al.	Original	Covid-19-Related Acute Invasive Fungal Sinusitis: Clinical Features and Outcomes	Infection (Fungal)	22	35096173
122	Alghonaim Y, Alfayez A, Alhedaithy R, et al.	Original	Recurrence Pattern and Complication Rate of Allergic Fungal Sinusitis: A 10-Year Tertiary Center Experience	Infection (Fungal)	28	33488733
123	McGhee NM, Groves S, Homan C, et al.	Case report	Allergic Fungal Rhinosinusitis in a 12-Year-Old Male Resulting in the Remodeling of Cribriform Plate With Protrusion Into the Anterior Cranial Fossa: A Case Report	Infection (Fungal)	1	38156154
124	El Hakkouni A, Harrar S, Hachimi A, et al.	Case report	Rhino-Orbito-Cerebral Mucormycosis: A Challenging Case	Infection (Fungal)	1	37809164
125	Al Nahwi FA, AlMomen A, Alkishi SA, et al.	Case report	The different clinical presentations of pediatric allergic fungal sinusitis	Infection (Fungal)	6	37096120
126	Babu K, Smitha KS, P Manjandavida F.	Original	Orbital Sarcoidosis in a High TB Endemic Country - A Case Series from South India	Infection (Sarcoidosis)	11	32073939
127	Xu-Yuan T, Hui-Yan L.	Case report	A rare ocular complication of septicemia: a case series report and literature review	Infection (Septicemia)	4	37558992
128	Shen MF, Chen HJ.	Case report	Unilateral proptosis in Lemierre's syndrome variant	Infection (Septicemia)	1	32546572
129	Gonzalez Barlatay J, Pagano Boza C, Hernandez Gauna GV, et al.	Review	Orbital Inflammation Caused by Aminobisphosphonates	Inflammation (Drug-induced)	43	35371416
130	Rivera Pérez de Rada P, Cobas Belso M, Vallinas Hidalgo S, et al.	Case report	Intraorbital rituximab to treat orbital inflammation in a patient with active neoplasia	Inflammation (IOI)	1	39732198
131	Peñarrocha-Oltra S, Balboa Miró M, Pérez-López M, et al.	Case report	Acquired enophthalmos in idiopathic orbital inflammatory disease	Inflammation (IOI)	1	38380084
132	Yilmaz Tugan B, Karabas L.	Case report	Idiopathic Orbital Inflammatory Disease Attack Associated with Large Serous Detachment Without Anterior and Posterior Segment Inflammation	Inflammation (IOI)	1	38089077
133	Macovei ML, Neacșu AM.	Case report	Diagnostic and therapeutic challenges in non-specific orbital inflammation - a case report	Inflammation (IOI)	1	37089801

134	Reshef ER, Freitag SK, Lee NG.	Case report	Orbital Inflammation Following COVID-19 Vaccination	Inflammation (IOI)	3	35323144
135	Pakdel F, Haghighi A, Pirmarzdashti N.	Case report	Disease modifying drugs in idiopathic sclerosing orbital inflammatory syndrome	Inflammation (IOI)	5	34030586
136	Chon BH, Hwang CJ, Perry JD.	Case report	Unilateral Ptosis, Proptosis, and Orbital Myositis After Shingles Vaccination	Inflammation (IOI)	1	33079763
137	Fang Y, Shen B, Dai Q, et al.	Review	Orbital inflammatory pseudotumor: new advances in diagnosis, pathogenesis, and treatment	Inflammation (Pseudotumor)	N/A	37794419
138	Kim JS, Blizzard S, Woodward JA, et al.	Original	Prostaglandin-Associated Periorbitopathy in Children and Young Adults with Glaucoma	Inflammation (Prostaglandin-associated periorbitopathy)	29	33008561
139	Chew LL, See W, Tan CL, et al.	Case report	Sight-Threatening Unilateral Posterior Scleritis With Positive Atypical p-ANCA As Early Manifestation of Lupus Spectrum Disease	Inflammation (Scleritis)	1	38765367
140	Savino G, Gambini G, Scorcia G, et al.	Case report	Orbital myositis and scleritis after anti-SARS-CoV-2 mRNA vaccines: A report of three cases	Inflammation (Scleritis)	3	36062603
141	Bennett K, Boccio E.	Case report	One in a Million: A Woman Presenting with Unilateral Painful Ophthalmoplegia	Inflammation (Tolosa-Hunt syndrome)	1	38869347
142	Ma Y, Lv K, Yang K, Wu H.	Case report	Secondary ocular hypertension due to tentorial dural arteriovenous fistula: a case report	Hemodynamics (AVF)	1	37697299
143	Kim SC, Kim JH, Kim CH, et al.	Case report	Middle temporal vein access for transvenous embolization of Cavernous sinus dural arteriovenous fistula: A case report and review of literature	Hemodynamics (AVF)	1	34592805
144	Lacorzana J, Rocha-de-Lossada C, Ortiz-Perez S.	Case report	A tricky case of unilateral orbital inflammation: carotid cavernous fistula in Graves-Basedow disease	Hemodynamics (CCF)	1	34179589
145	Kısabay Ak A, Çınar C, Doğan GN, et al.	Original	Clinical improvement in indirect carotid cavernous fistulas treated endovascularly: A patient based review	Hemodynamics (CCF)	18	34146840
146	Peterson E, Minalyan A, Downey C.	Case report	Carotid-Cavernous Fistula in a Patient With Unilateral Periorbital Swelling and Proptosis Initially Attributed to Possible Immunoglobulin G4-Related (IgG4-Related) Disease	Hemodynamics (CCF)	1	37384089
147	Cosentino G, Comi S, Maglionico MN, et al.	Case report	A case of carotid cavernous fistula mimicking Graves' orbitopathy	Hemodynamics (CCF)	1	37347382
148	Giragani S, Kasireddy AR, Agrawal V, et al.	Case report	Unilateral Basal Ganglia Hyperintensity Secondary to Venous Congestion in a Case of Indirect Carotico-cavernous Fistula	Hemodynamics (CCF)	1	34507443
149	Gasparian SA, Chalam KV.	Case report	Successful repair of spontaneous indirect bilateral carotid-cavernous fistula with coil embolization	Hemodynamics (CCF)	1	33927873
150	Salim S, Koka K, Halbe S, et al.	Case report	Superior ophthalmic vein thrombosis post manual carotid compression for indirect carotid-cavernous fistula	Hemodynamics (CCF)	5	33032482
151	Boukili K, Elmaaloum L, Allali B, et al.	Case report	Unilateral exophthalmia revealing large aneurysm of the internal carotid: a case report	Hemodynamics (Intracavernous artery aneurysm)	1	34603577
152	Ramakrishnan A, Singh	Case report	A Case Report on Acute Visual Loss With Ophthalmoplegia Following Spine	Hemodynamics	1	39360104

	S, Puri SK.		Surgery	(Postoperative CRA occlusion)		
153	Pichayawat C, Chokthaweesak W, Leelawongs S, et al.	Case report	Acute unilateral orbital varix thrombosis in preexisting bilateral orbital varices: illustrative case	Hemodynamics (Thrombosis)	1	37354429
154	Gietzelt C, Wiedemann J, Lappas A, et al.	Case report	Thrombosis of the superior ophthalmic vein-A case series and review of the literature	Hemodynamics (Thrombosis)	3	39266751
155	Rangappa R, Deshpande R, Teja ESP, et al.	Case report	Cerebral venous sinuses thrombosis post extracorporeal membrane oxygenation: a case report	Hemodynamics (Thrombosis)	1	38919201
156	Vigneul E, Van Boxstael E, Goffette P, et al.	Case report	Unilateral proptosis and jugular thrombosis following ventriculoperitoneal shunt placement	Hemodynamics (Thrombosis)	1	38424399
157	Abdelsalam A, Ramsay IA, Ehiemua U, et al.	Case report	Thrombosed orbital varix of the inferior ophthalmic vein: A rare cause of acute unilateral proptosis	Hemodynamics (Thrombosis)	1	37404515
158	Gómez-Arbeláez D, García-Gutiérrez A, González-Fajardo JA.	Case report	Unilateral Proptosis (Exophthalmos) Caused by Axillary-Subclavian Venous Thrombosis in a Patient with Upper Extremity Arteriovenous Dialysis Fistula	Hemodynamics (Thrombosis)	1	34824675
159	Ali S.	Case report	Cavernous Sinus Thrombosis: Efficiently Recognizing and Treating a Life-Threatening Condition	Hemodynamics (Thrombosis)	1	34567880
160	Agha A, Pangy T, Ajayi O, et al.	Case report	Septic cavernous sinus thrombosis; A rare cause of unilateral exophthalmos	Hemodynamics (Thrombosis)	1	33093709
161	Sheth NT, Lee IT, Woodward JA, et al.	Case report	Nontraumatic orbital hematoma secondary to labor and childbirth: a case report and review of the literature	Hemodynamics (Vascular malformation)	1	38668873
162	Gabbard RD, Dryden SC, Reggie SN, et al.	Case report	Spontaneous orbital hemorrhage in a case of acute liver failure	Hemodynamics (Vascular malformation)	1	35434419
163	Köksoy ÜC, Yilmaz H, Kazbek BK, et al.	Case report	An Eye-Popping Experience Immediately After Intubation: A Case Report	Hemodynamics (Vascular malformation)	1	34550908
164	Issiaka M, Jamaleddine H, El Belhadji M, et al.	Case report	Orbital varix: A rare case of unilateral exophthalmos, case report	Hemodynamics (Vascular malformation)	1	34026106
165	Różańska-Walędziak A, Szewczuk O, Walędziak M, et al.	Case report	Spontaneous unilateral exophthalmos after a vaginal delivery	Hemodynamics (Vascular malformation)	1	32279535
166	Raicevic M, Nikolovski SS, Nedovic S, et al.	Case report	Unilateral Exophthalmos as the First Sign of Chronic Obstructive Hydrocephalus in a Pediatric Patient: A Case Report	Miscellaneous (Chronic obstructive hydrocephalus)	1	39553149
167	Kwok IUK, Sakinah Z, Elmina M, et al.	Case report	Ectopic lacrimal gland causing intermittent proptosis	Miscellaneous (Ectopic lacrimal gland)	1	35399977
168	Nagendran S, Alsamnan	Review	Ectopic Lacrimal Gland Tissue: A Systematic Review	Miscellaneous	180	32205779

	M, Strianese D, et al.			(Ectopic lacrimal gland)		
169	Cutting S, Davies-Husband C, Poitelea C.	Case report	Recurrent Self-Induced Nontraumatic Orbital Emphysema Causing Orbital Compartment Syndrome with Optic Nerve Dysfunction	Miscellaneous (Nontraumatic orbital emphysema)	1	33777467
170	Wu CM, Liao HE, Hsu SW, et al.	Case report	Cervicogenic exophthalmos: Possible etiology and pathogenesis	Miscellaneous (Sympathetic dystrophy)	1	32047780
171	Paul S, Tost F, Hübner BC.	Case report	Unilateral Exophthalmos	Miscellaneous (Paget's disease)	1	36507730

No., list number; N/A, not applicable

TAO, thyroid-associated ophthalmopathy; CCF, carotid-cavernous fistula; ECIO, external compressive ischemic orbitopathy; DSRCT, desmoplastic small round cell tumor; LCH, langerhans cell histiocytosis; NET, neuroendocrine tumor; IOI, idiopathic orbital inflammatory disease; AVF, arteriovenous fistula; CRA, central retinal artery
